# 127aa encoded by circSpdyA promotes FA synthesis and NK cell repression in breast cancers

**DOI:** 10.1038/s41418-024-01396-1

**Published:** 2024-10-14

**Authors:** Xinya Gao, Zicheng Sun, Xin Liu, Jiayue Luo, Xiaoli Liang, Huijin Wang, Junyi Zhou, Ciqiu Yang, Tiantian Wang, Jie Li

**Affiliations:** 1 Department of Breast and Thyroid Surgery, Guangzhou Women and Children’s Medical Center, Guangzhou, Guangdong 510080 China; 2 Institute of Reproductive Health and Perinatology, Guangzhou Women and Children’s Medical Center, Guangzhou Medical University, Guangzhou, 510623 P.R. China; 3 Breast Disease Center, The First Affiliated Hospital of Sun Yat-sen University, Guangzhou, Guangdong 510080 China; 4 Department of Breast Cancer, Guangdong Provincial People’s Hospital (Guangdong Academy of Medical Sciences), Southern Medical University, Guangzhou, Guangdong 510000 China; 5 Department of Thyroid Surgery, The Second Affiliated Hospital of Zhejiang University College of Medicine, Hangzhou, Zhejiang 310009 China

**Keywords:** Cancer metabolism, Cancer microenvironment

## Abstract

Lipid metabolism reprogram plays key roles in breast cancer tumorigenesis and immune escape. The underlying mechanism and potential regulator were barely investigated. We thus established an in vivo tumorigenesis model, mice-bearing breast cancer cells were treated with an ordinary diet and high-fat diet, species were collected and subjected to circRNA sequence to scan the potential circRNAs regulating the lipid metabolism. CircSpdyA was one of the most upregulated circRNAs and had the potential to encode a 127-aa micro peptide (referred to as 127aa). 127 aa promotes tumorigenesis through promoting the fatty acid de novo synthesis by directly binding to FASN. Single-cell sequence indicated 127aa inhibited NK cell infiltration and function. This was achieved by inhibiting the transcription of NK cell activators epigenetically. Moreover, lipid-laden from 127aa positive cancer cells transferred to NK cells inhibited the cytotoxicity. Taken together, circSpdyA encoded 127aa promotes fatty acid de novo synthesis through directly binding with FASN and induced NK cell repression by inhibiting the transcription of NK cell activators.

## Introduction

Clinicians face challenges in the treatment of triple-negative breast cancer (TNBC) because TNBC cells do not express specific proteins, namely, estrogen receptor (ER), progesterone receptor (PR), and human epidermal growth factor receptor 2 (HER2). However, recent studies have shown that TNBC is characterized by heterogeneity in transcriptomic and metabolic pathways. TNBC is classified into four subtypes according to their transcriptomes: luminal androgen receptor (LAR), immunomodulatory (IM), basal-like immune-suppressed (BLIS), and mesenchymal-like (MES) TNBC [1]. Additionally, TNBC is classified into three metabolic pathway-based subtypes (MPSs): MPS1, defined as the lipogenic subtype; MPS2, defined as the glycolytic subtype; and MPS3, defined as the mixed subtype [2]. Groundbreaking advancements in TNBC typing are crucial for identifying potential therapeutic targets of TNBC. Reactivation of fatty acid (FA) synthesis is a critical feature of tumor cell growth and survival because FAs serve as energy sources, regulate membrane structures, and function as signaling molecules [3]. Increased lipid metabolism has been shown to be important for TNBC, especially in combination with sensitivity to ferroptosis [4, 5]. Yang et al. reported that TNBC cells exhibit heterogeneity in ferroptosis and that ferroptosis-related metabolic mechanisms are enhanced in the LAR subtype [5]. GSH peroxidase 4 (GPX4) was demonstrated to be a hallmark gene, and GPX4 inhibitors in combination with immunotherapy improve the prognosis of patients with the LAR subtype. Researchers have also shown that FA metabolism is a hallmark metabolic pathway in LAR TNBC [5]. However, the precise factors that regulate FA synthesis and the underlying molecular mechanisms have not been further explored.

In the past decade, the pivotal roles of circular RNAs (circRNAs) in tumorigenesis and progression have been demonstrated. Some circRNAs perform their biological functions by encoding specific peptides or proteins [6]. Studies have shown that specific proteins encoded by circRNAs can promote TNBC cell proliferation, apoptosis, migration, and invasion. Yang et al. confirmed that the FBXW7-185aa protein encoded by circFBXW7 can suppress TNBC progression [7]. Li et al. discovered that circ-HER2 encodes the protein HER2–103, which sustains TNBC tumorigenicity by interacting with and activating EGFR/HER3 and promoting AKT phosphorylation [8]. Accumulating evidence has revealed the regulatory role played by circRNAs in cancer metabolism. The expression and activity of many key regulators involved in lipid metabolism, such as AMP-activated protein kinase (AMPK), fatty acid synthase (FASN), and acetyl-CoA carboxylase (ACC), are associated with energy reserves and rapid proliferation of tumor cells [9]. Several circRNAs participate in the lipid metabolic reprogramming of tumor cells by activating and regulating the AMPK complex, driving de novo FA synthesis, and altering the cellular distribution of FASN mRNA [10–12]. However, it is still unclear whether circRNAs regulate FA synthesis in TNBC despite their potential role in lipid metabolism reprogramming.

Metabolically active cancer cells can cause nutrient depletion and hypoxia and generate an acidic, toxic metabolite-enriched tumor microenvironment (TME) that suppresses antitumor immunity. Increased FA synthesis provides essential energy sources and regulatory factors for cancer cells. It also upregulates exogenous FA uptake. Ultimately, intracellular FA accumulation can modify the tumor-promoting phenotype of immune cells [13]. Natural killer (NK) cells are important members of the innate immune system. In the context of cancer, NK cells are presumed to be directly activated without antigen-presenting processes and to exert cytotoxic effects on tumor cells. Unfortunately, NK cell responses are impaired in the TME owing to immunosuppressive byproducts generated by abnormal lipid metabolism [14]. Lipid accumulation diminishes the antitumor capacity of NK cells, which mainly results in NK cell dysfunction [3]. A previous study showed that NK cells are likely the main cell type in the TME that are involved in immunosuppression and determining the aggressiveness of TNBC tumors [15]. Given that lipid metabolism reprogramming is a feature of TNBC and impacts NK cell function in the TME, the interaction between lipid metabolism and NK cells in TNBC is worth elucidating.

In this study, we transplanted the MDA-MB-231 TNBC cell line into immunocompromised mice and fed the mice an ordinary diet or a high-fat diet. Then, the tumors were collected and subjected to circRNA sequencing, and circSpdyA (hsa_circ_0053332) was found to be the most dysregulated circRNA. A subsequent bioinformatical analysis revealed that circSpdyA has the potential to encode a 127-aa protein, and this finding was confirmed by LC-MS and immunoblotting. This protein, referred to as 127aa, directly promotes fatty acid synthesis by binding to FASN. Next, we used single-cell sequencing to reveal the potential biological function of 127aa in cancer immunity, and the results suggested that additional fatty acid storage inhibited the recruitment and function of NK cells by inhibiting the transcription of NK cell-activating factors, such as ULBP2 and MICA.

## Results

### circSpdyA is a potential regulator of fatty acid metabolism in TNBC

**Fig. 1:**
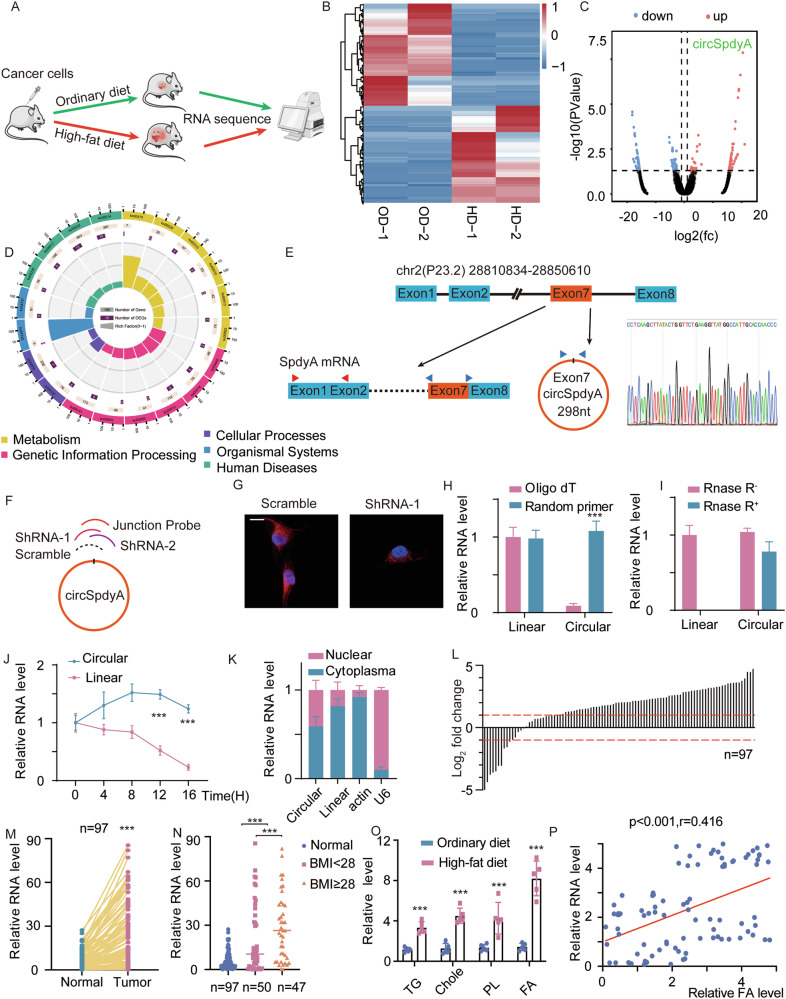
circSpdyA was a potential regulator of lipid metabolism. **A** Graphic illustration of circRNA sequence for potential fat-mediated tumorigenesis, mice bearing MDA-MB-231 cells were treated with ordinary diet and high-fat diet. Tumor speciesmens were collected and subjected to circRNA sequence. **B** The heatmap of the dysregulated circRNAs, OD, ordinary diet, HD, high-fat diet. **C** The volcano of the dysregulated circRNAs. circSpdyA was indicated, X-axis, Log2 (FC), Y-axis, -Log10 (P value). **D** The GO annotation of the source gene of dysregulated circRNAs. **E** The illustration of circSpdyA and linear SpdyA, the sanger sequence of products spanned by junction specific probes. **F** The illustration of junction-specific shRNA and probe. **G** FISH of circSpdyA in cells with junction-specific shRNAs. **H** The amplifications of linear and circular SpdyA using Oligo dT and random primer. **I** RNAs were extracted and subjected to Rnase R digestion, and linear and circular RNA was detected. **J** The half-life time of linear and circular SpdyA in MDA-MB-231 cells. **K** Different cell fraction was collected and subjected to qRT-PCR, linear and circular SpdyA was detected, and actin and U6 were applied as positive control. **L** The log2 fold change of circSpdyA in 97 TNBC samples. **M** The relative expression level of circSpdyA in 97 paired TNBC and paired normal tissues. **N** 97 TNBC patients were divided into two groups according to the BMI value. The relative circSpdyA level was determined. **O** The relative level of TG, Chole, PL, and FA in tumors derived from A. TG, triglyceride; PL, phospholipid; FA, fatty acid. **P** The regression analysis of circSpdyA and FA level in 89 TNBC samples.

Three subtypes of TNBC are characterized according to their metabolic pathways: lipogenic, glycolytic, and mixed [2]. To identify potential circRNAs that regulate lipid metabolism in TNBC, we collected tumor tissues and performed circRNA sequencing. Mouse models of TNBC were established by the injection of TNBC cells, and the mice were divided into two groups and fed an ordinary diet (OD) or high-fat diet (HD) (Fig. 1A). There were 116 upregulated circRNAs and 120 downregulated circRNAs in tumor tissues from the HD group compared with tumor tissues from the OD group (p < 0.05 and fold change > 1; Fig. 1B). Among the differentially expressed circRNAs, circSpdyA (hsa_circ_0053332) was the most highly upregulated circRNA in the HD group (Fig. 1C). Kyoto Encyclopedia of Genes and Genomes (KEGG) enrichment analysis suggested that the differentially expressed genes were enriched in pathways such as ‘Metabolism, Organismal Systems, and Genetic Information Processing’ (Fig. 1D). Based on these results, we focused on circSpdyA as the target circRNA. We then utilized a convergent primer for SpdyA mRNA and a divergent primer spanning the junction site of circSpdyA to validate the sequence of this gene. Sanger sequencing suggested that circSpdyA was generated from exon 7 of *SpdyA* with a 298 nt spliced sequence length. (Fig. 1E). To confirm the cellular location of circSpdyA, we designed a junction probe to detect endogenous circSpdyA and two specific paring junction shRNAs (referred to as sh1 and sh2) to decrease circSpdyA expression (Fig. 1F). After MDA-MB-231 cells were transfected with these shRNAs, we observed by FISH that circSpdyA was mostly located in the cytoplasm. shRNA-1 could specifically knock down the expression of circSpdyA (Fig. 1G). Due to the covalently closed loop structure and lack of a poly(A) tail of circRNAs, circSpdyA could be specifically amplified with random primers rather than oligo(dT) primers (Fig. 1H). Compared with their linear counterparts, circRNAs are relatively stable. Consequently, circSpdyA was resistant to RNase R digestion according to RT‒qPCR analysis (Fig. 1I). The half-life of circSpdyA was also longer than that of linear SpdyA (Fig. 1J). A cell fraction qPCR assay confirmed that more circSpdyA was present in the cytoplasm than in the nucleus (Fig. 1K). We collected 97 tumors and paired normal tissues from TNBC patients. RT‒qPCR analysis suggested increased expression of circSpdyA in tumor tissues (Fig. 1L, M), indicating that circSpdyA may be specifically upregulated in TNBC cells. To further investigate whether the expression of circSpdyA is relevant to lipid metabolism, we divided the TNBC patients into two groups based on their body mass index (BMI). Analysis of the BMI ≥ 28 groups showed that the mean expression of circSpdyA was significantly greater than that in the BMI < 28 group and normal tissue samples (Fig. 1N). Additionally, we determined the content of different lipid components in tumor tissues from the OD and HD groups, as described in Fig. 1A. Fatty acids were the most significantly upregulated lipids in tissues from the group (Fig. 1O), suggesting that FA metabolism was the most dysregulated lipid reprogramming pathway in TNBC tissues of mice that were fed a high-fat diet. We hypothesized that circSpdyA contributes to cancer progression primarily by affecting FA metabolism. Next, regression analysis of circSpdyA and FA levels revealed a positive correlation in tumor samples (Fig. 1P). These results suggested that circSpdyA is specifically upregulated in TNBC tissues and may regulate FA metabolism in tumors.

### Identification of 127aa encoded by circSpdyA

**Fig. 2:**
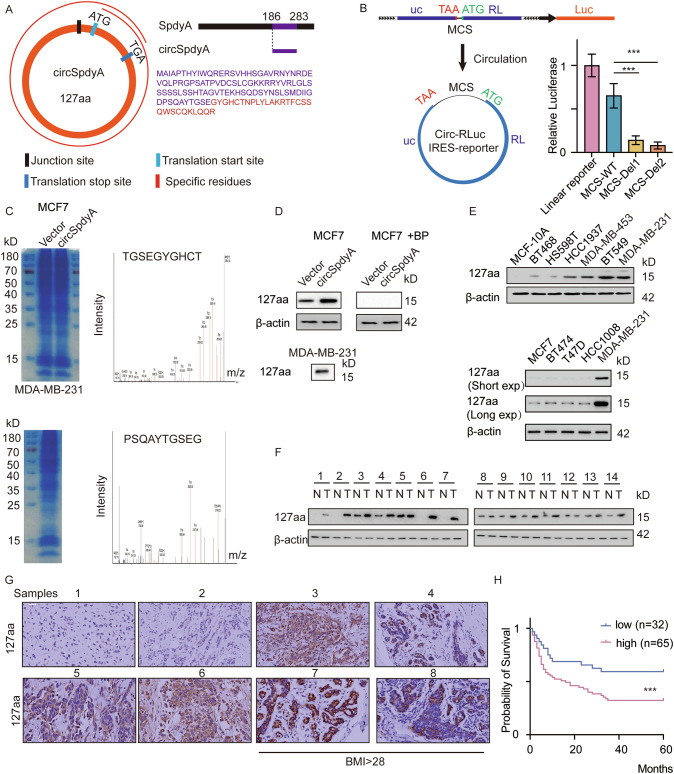
circSpdyA encoded a micro peptide 127aa. **A** Graphic illustration of circSpdyA and encoded micro peptide, the junction site and translation start/stop site, and the specific residues were indicated. **B** The illustration of the circular IRES reporting system and the relative luciferase activity of indicated alleles. **C** The coomassie blue staining of the whole cell lysate of indicated cells. The specific residues were detected using LC-MS. **D** 127aa specific antibody was generated, and 127aa was detected in cells with indicated modifications. **E** The immunoblot of 127aa in TNBC cells and non TNBC cells. **F** The immunoblot of 127aa in 7 paired TNBC samples and normal tissues. **G** The IHC of 127aa in 8 TNBC species; 1-6, BMI < 28; 7-8, BMI > 28. **H** The overall survival analysis of 97 TNBCs according to the IHC score obtained from (**F**).

Extensive research has shown that circRNAs can be effectively translated into detectable peptides that have biological functions in cancer [6]. Two critical elements determine the mechanism of circRNA translation: the internal ribosome entry site (IRES), which directly recruits ribosomes to initiate translation, and open reading frames (ORFs), which encode peptides [16]. Upon inspection of the circSpdyA sequences, an ORF was identified that could encode a novel 127-amino acid peptide(127aa). We mapped homologous coding regions in SpdyA mRNA and circSpdyA and observed that 127aa had an additional 10 amino acid-long region in its tail, which was absent in the SpdyA mRNA-encoded protein (Fig. 2A). Then, we utilized a circular vector-based luciferase assay to identify the IRES sequence of circSpdyA (Fig. 2B). Hence, we assumed that circSpdyA can encode a protein. To validate whether circSpdyA was translated, we generated an overexpression circSpdyA vector and then transfected the overexpression and negative control vectors into MCF7 cells. We also generated an antibody specific for 127aa to detect its expression from the vector in MCF7 cells via liquid chromatography-mass spectrometry (LC-MS) (Fig. 2C, up). We found that 127aa was endogenously expressed in MDA-MB-231 cells (Fig. 2C, down). LC-MS analysis was further performed to determine the specific amino acid residues (EG). We further confirmed the specificity of the antibody by using the specific antibody and by transfecting a 127aa blocking peptide (BP) into MCF7 cells (Fig. 2D). The expression of 127aa was shown in several established TNBC cell lines; 127aa was upregulated in breast cancer cell lines, weakly expressed in MCF-7 cells, and not expressed in MCF-10A cells, for further confirmation, no-TNBC cells such as MCF-7, BT474, T47D, HCC1008 was separately detected, and immunoblot with short/long exposure time was obtain (MDA-MB-231 as positive control), the expression of 127aa in non-TNBCs was negligible compared with MDA-MB-231 (Fig. 2E), indicating that its expression was specific upregulated to TNBC cells. Moreover, 127aa was detected in TNBC samples using immunoblotting and IHC assays. 127aa was upregulated in tumors compared with adjacent normal tissues (Fig. 2F). Then, patients were divided into two groups according to their BMI, and the expression of 127aa was dramatically greater in patients with a BMI > 28 (Fig. 2G). We monitored survival outcomes in an in-house cohort of 97 TNBC patients and noted that high expression of 127aa was correlated with poor overall survival (Fig. 2H). Overall, a novel protein, 127aa, was confirmed to be specifically upregulated in TNBC cells, and its expression suggested the possibility of poor outcomes in TNBC patients.

### 127aa promotes TNBC tumorigenesis in vitro and in vivo

**Fig. 3:**
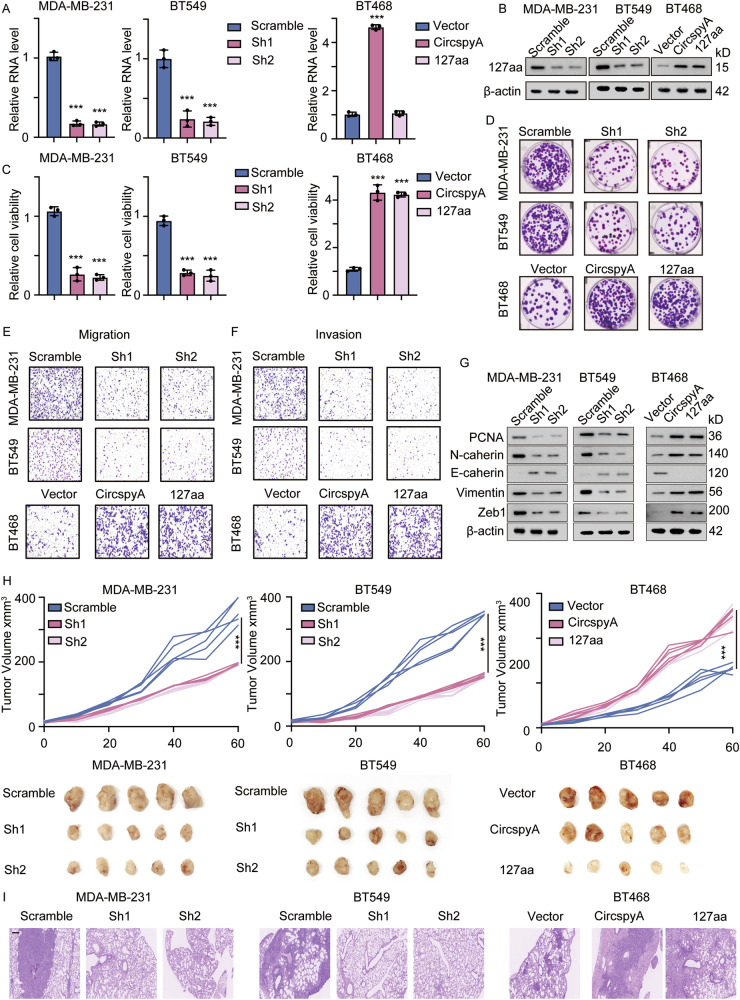
127aa promotes proliferation, migration, and invasion of TNBC cells in vivo and in vitro. **A** qPCR detecting circSpdyA in different cell lines. **B** Immunoblot of 127aa in indicated cell lines. **C** The relative cell viability detected by CCK-8 assay in circSpdyA knocking down cells and overexpression cells. **D** The colony formation assay of cells with indicated modifications. **E** The representative image of transwell assay in different cells. **F** The representative image of invasion chamber assay in different cells. **G** The immunoblot of PCNA and EMT markers in different cells. **H** Upper: Different cancer cells were transplanted into immunocompromised mice, and the tumor volume was detected and analyzed. Lower: The final picture of the xenograft models of mice bearing indicated cancer cells. **I** The representative image of HE staining of the lungs from mice bearing different cancer cells.

Hypoxia and nutrient deprivation in the TME cause rapid changes in metabolic reprogramming and immune suppression. Tumor cells increase the translation of specific circRNAs under these conditions to support their survival and acquired resistance to tumor immunity [17]. We speculated that 127aa could promote the tumorigenesis and proliferation of TNBC cells. We first established different TNBC cell lines with stable circSpdyA knockdown by using sh1 and sh2 as previously described as well as BT468 cell lines with stable circSpdyA and 127aa overexpression. qPCR and immunoblotting were subsequently performed (Fig. 3A, B). Decreased expression of circSpdyA inhibited TNBC cell proliferation, migration, and invasion, as measured by cell viability, colony formation, migration, and invasion assays. As expected, overexpression of circSpdyA and 127aa promoted TNBC tumorigenesis (Fig. 3C–F). Immunoblotting was used to measure the expression of epithelial-to-mesenchymal transition (EMT) markers and a proliferation marker (PCNA) in cells with the indicated modifications. The results indicated that 127aa promoted mesenchymal transition, as shown by elevated expression of N-cadherin, vimentin, and Zeb1 (Fig. 3G). To reveal the biological importance of 127aa in TNBC tumorigenesis and progression in vivo, we next generated xenograft and lung metastasis mouse models. The mice were transplanted with different cancer cells, and tumor volume was detected and analyzed. Tumour growth was inhibited in the circSpdyA-knockdown group, whereas it was accelerated in the circSpdyA- and 127aa-overexpressing groups (Fig. 3H, Supplementary Fig. 1A). HE staining was used to detect lung metastasis colonies. Circulating tumor cells were also detected and normalized to monocyte levels. The numbers of lung metastasis colonies and circulating tumor cells were decreased in mice bearing circSpdyA-knockdown cells and increased in 127aa-overexpressing xenograft models (Fig. 3I, Supplementary Fig. 1B).

### 127aa promotes fatty acid synthesis and fatty acid oxidation

**Fig. 4:**
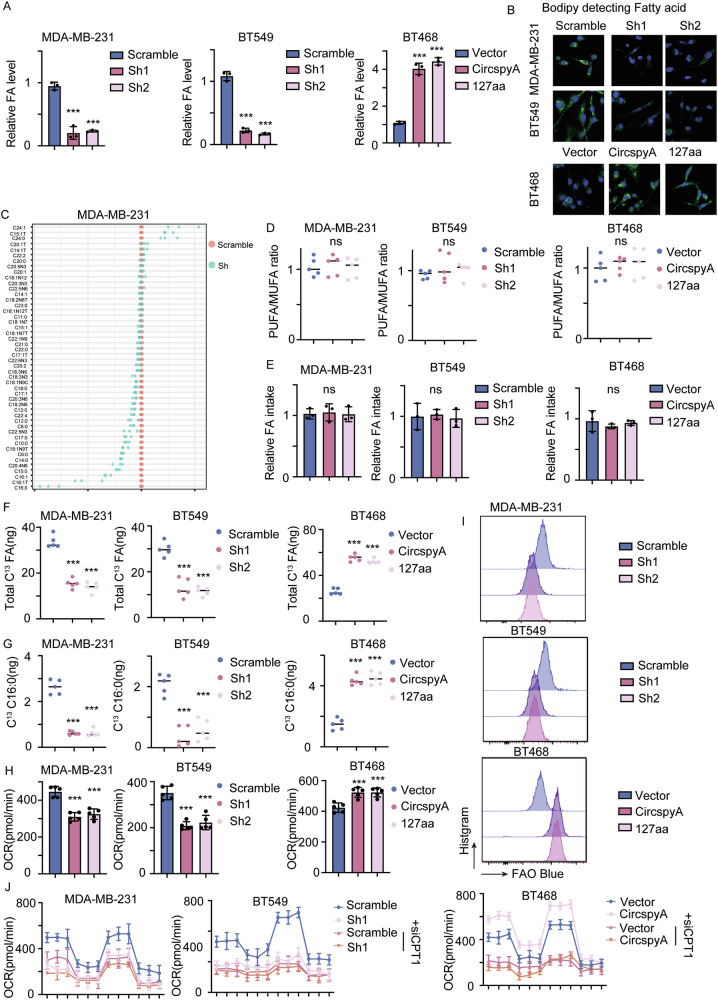
127aa promotes the synthesis and oxidation of fatty acid. **A** The total fatty acid level in cells with indicated modifications. **B** Cells were stained with Bodipy detecting fatty acid, the representative images were obtained using the confocal microscope. **C** circSpdyA knocking down cells were collected and subjected to fatty acid metabolome analysis, and the relative level of different fatty acids was determined. **D** Polyunsaturated fatty acid (PUFA) and monounsaturated fatty acid (MUFA) were detected, and the ratio was determined and analyzed. **E** Cells were incubated with FITC-labeled fatty acid, intracellular FITC-labeled fatty acid was detected and FA intake rate was calculated. **F** Cells were incubated with C^13^-labeled glucose and C^13^-labeled total FA was detected. **G** C16:0 fatty acid was the firstly synthesized fatty acid. C^13^-labeled C16:0 was detected. **H** The oxygen consumption rate was detected in different cell lines. **I** Cancer cells were treated with FAO Blue and subjected to FACS detecting FAO activity in cells with indicated modifications. **J** CPT1 was the limiting enzyme for the oxidation of fatty acid, cells were transfected with CPT1-specific siRNA and subjected to OCR assay.

We previously demonstrated that circSpdyA and 127aa were correlated with obesity and FA levels (Fig. 1N–P). To determine the mechanism by which 127aa controls FA maintenance, we first determined the total FA levels in different cells. As expected, the total FA content was decreased in circSpdyA-knockdown cell lines but increased in circSpdyA- and 127aa-overexpressing cells (Fig. 4A). This was further confirmed by the use of a BODIPY lipid detection probe (Fig. 4B). Fatty acid metabolomics analysis was performed to determine FA heterogeneity in circSpdyA-knockdown cell lines. The total FA levels were decreased, which was expected since the imbalance between polyunsaturated fatty acids (PUFAs) and monounsaturated fatty acids (MUFAs) is crucial for cell survival. We next determined the PUMA/MUFA ratio. Knocking down circSpdyA did not alter the balance between PUFAs and MUFAs (Fig. 4C, D). Given that FA levels are maintained by FA intake from the extracellular matrix or by de novo fatty acid synthesis, we incubated cancer cells with FITC-labelled fatty acids and measured the intracellular levels of FITC-labelled fatty acids. FA intake was thus determined and analyzed. The results demonstrated no difference in FA intake between the different cell lines (Fig. 4E). ^13^C-labelled glucose was added and degraded into ^13^C-acetyl-CoA, and FAs that were newly synthesized from acetyl-CoA were thus labelled with 13 C. Similarly, the relative levels of ^13^C-labelled total FAs and C16:0 FAs were decreased in circSpdyA-knockdown cells, indicating that FA de novo synthesis was dramatically inhibited (Fig. 4F, G). FAO is the main pathway of FA metabolism and provides energy in the nutrient-deficient tumor environment. The oxygen consumption rate (OCR) was measured to analyze the FAO rate. The OCR was inhibited in circSpdyA-knockdown cells and enhanced in circSpdyA- and 127aa-overexpressing cells (Fig. 4H). FA specific probe FAO blue was applied to further detect the oxidation of fatty acid, as expected, 127aa promotes the oxidation of fatty acid (Fig. 4I). Carnitine palmitoyltransferase I (CPT1) is the key rate-limiting enzyme of FAO. We next knocked down CPT1 with a specific siRNA and subjected the cells to an OCR assay. Knocking down CPT1 inhibited the OCR of cancer cells and completely reversed the 127aa-mediated promotion of FAO (Fig. 4J). These results further confirmed that circSpdyA and 127aa promote the FAO of intracellular FAs.

### 127aa directly binds to FASN and promotes FASN-mediated de novo FA synthesis

**Fig. 5:**
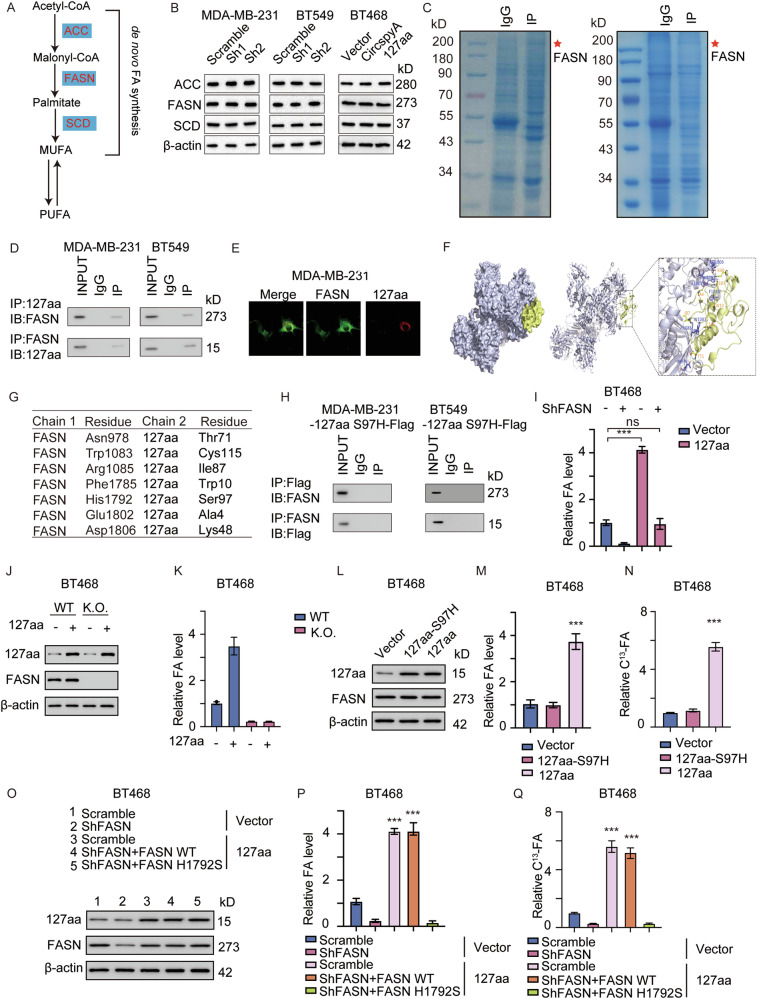
127aa directly binds with FASN. **A** The illustration of de novo synthesis of fatty acid. **B** Immunoblot of the key enzymes of fatty acid synthesis. **C** The coomassie blue staining of the co-IP complex using 127aa antibody in MDA-MB-231 and BT549 cells. **D** The IP-IB assay detects the interaction between 127aa and FASN. **E** The IF staining of 127aa and FASN in MDA-MB-231 cells. **F** Molecular docking predicting the interaction between FASN and 127aa. **G** Interacting residues from FASN and 127aa predicted on (**F**). **H** 127aa mutant allele was designed and transfected into MDA-MB-231 cells, and IP-IB assay was applied to detect FASN and Flag. **I** Total fatty acid level in cells with indicated modifications. **J** FASN was knocked out using in BT468 cells and 127aa was overexpressed, respectively. Immunoblot was applied detecting FASN and 127aa. **K** Total fatty acid level in cells described in (**J**). **L** 127aa and 127aa mut was overexpressed in BT468, respectively. Immunoblot detecting 127aa and FASN. **M** Total fatty acid level in cells described in (**L**). **N** Total C^13^ labelled fatty acid level in cells described in (**L**). **O** FASN was knocked down using shRNAs and WT/H1792S mutant allele was re-expressed, 127aa was then overexpressed. Immunoblot was applied detecting FASN and 127aa. **P** Total fatty acid level in cells described in (**O**). **Q** Total C^13^ labelled fatty acid level in cells described in (**O**).

MUFAs are synthesized from acetyl-CoA through the interaction of ACC, FASN, and stearyl-CoA desaturase (SCD) (Fig. 5A). We hypothesized that circSpdyA-mediated FA maintenance was accomplished by upregulating the expression of the limiting enzyme or enhancing its enzymatic activity. Thus, we first measured the expression of ACC, FASN, and SCD in circSpdyA-knockdown and circSpdyA-overexpressing cells, and no significant differences were found (Fig. 5B). To determine the direct and indirect regulatory mechanisms involved, we collected MDA-MB-231 and BT549 cells and subjected them to co-IP. FASN was the hub of the protein-interacting network (Fig. 5C), indicating that 127aa may directly bind to FASN. This was further confirmed by IP-IB and IF (immunofluorescence) assays (Fig. 5D, E). Furthermore, we performed a molecular docking analysis of FASN to characterize the interaction motif in FASN and the binding sites in 127aa (Fig. 5F, G). To validate this interaction, we constructed the 127aa motif mutant by changing the interacting residue (S97H), and then, we observed that mutation of the 127aa binding site disrupted the interaction between FASN and 127aa (Fig. 5H). To determine the biological importance of 127aa in FASN and FA maintenance, we first knocked down FASN in 127aa-overexpressing cells. Knocking down FASN decreased the FA levels and reversed 127aa-mediated FA synthesis (Fig. 5I). For further confirmation, we next knocked FASN out and overexpressed 127aa, respectively. Relative fatty acid was thus detected. 127aa promotes the level of fatty acid, but in FASN K.O. cells, overexpression of 127aa have no effect on the overall fatty acid level (Fig. 5J, K), indicating that 127aa exert its’ function in FASN dependent manner. We next transfected 127aa-WT or 127aa-S97H into BT468 cells, after which the intracellular FA level and newly synthesized FA level were measured. The 127aa mutant failed to increase the total and newly synthesized FA level (Fig. 5L–N). Finally, we knocked down FASN and re-expressed the WT and H1792S mutant FASN proteins in 127aa-overexpressing cells, after which the intracellular FA level was measured. In 127aa-overexpressing cells, only WT FASN but not mutant FASN promoted FA accumulation (Fig. 5O–Q).

### 127aa inhibits NK cell infiltration and function

**Fig. 6:**
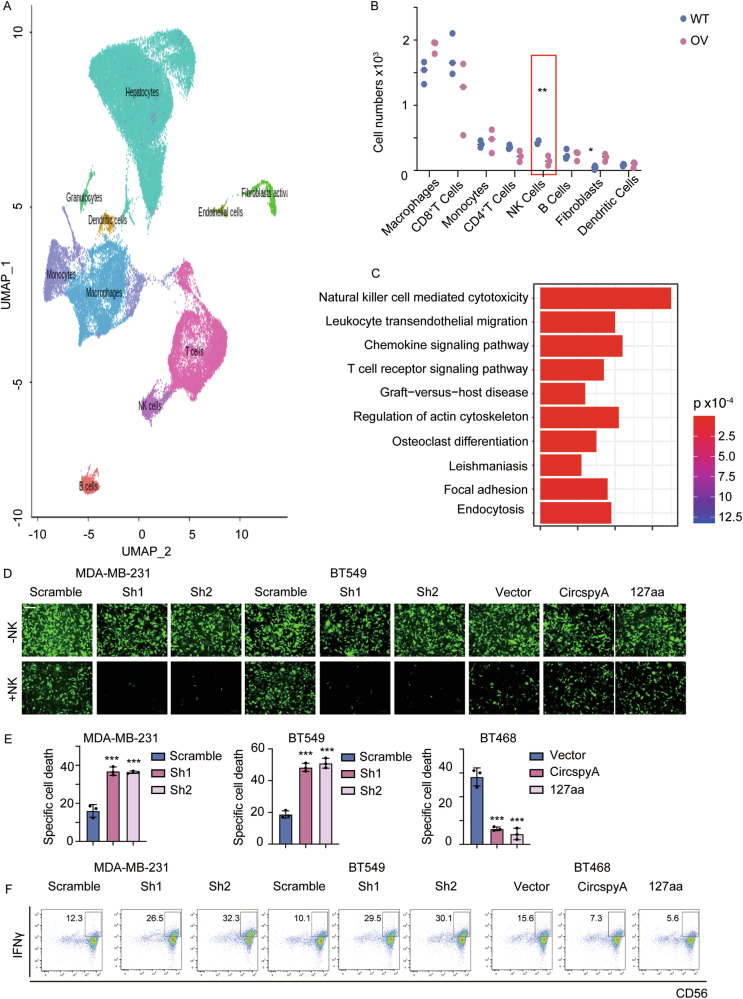
127aa inhibits the infiltration and function of NK cells. **A** Mice breast cancer cells 4T1 were transfected with 127aa and were then transplanted into C57 mice, tumors were collected and subject to single cell sequence, and the UMAP of the scRNA sequence was generated. **B** The number of infiltrated cells per 10^3^ cells. **C** The dysregulated pathways in NK cells derived from WT and 127aa OV tumors. **D** Indicated cancer cells were co-cultured with NK cells and then the Calcein-AM cell killing assay was applied, and the representative image was captured. **E** The statistical analysis of specific cell death in (**D**). **F** Flow cytometry analyzing the IFNγ^+^ proportion NK cells described in (**D**).

Obesity and FA levels play key roles in innate immunity and adaptive immunity [18, 19]. To determine the potential function of 127aa in cancer-associated immunity, we established 127aa-overexpressing mouse 4T1 breast cancer cell lines and then subcutaneously transplanted them into C57BL/6 J mice. Single-cell RNA sequencing (scRNA-seq) was performed on immune cells isolated from tumor tissues (Fig. 6A). According to the scRNA-seq data, the immune cell clusters were categorized as macrophages, monocytes, CD8+ and CD4 + T cells, NK cells, B cells, fibroblasts, and dendritic cells. Among these clusters, significantly fewer NK cells were observed in the 127aa-overexpressing group than in the WT group, which had relatively more NK cells (Fig. 6B). Transcriptome enrichment analysis revealed that immune cells were significantly enriched in the natural killer cell-mediated cytotoxicity signaling pathway, and other enriched signals included those related to the ‘chemokine signaling pathway and ‘regulation of the actin cytoskeleton’, which are critical for NK cell recruitment and function (Fig. 6C). We cocultured different BC cell lines with NK cells isolated from human peripheral blood. Compared to the environment without NK cells, the survival rates of BC cells in the presence of NK cells were lower. In the cocultured microenvironment, knocking down circSpdyA resulted in increased BC cell death, which was partially reversed by the overexpression of circSpdyA and 127aa (Fig. 6D, E). Compared to that in the scramble group, the activity of NK cells in the microenvironment was increased in the circSpdyA knockdown group, whereas the activity of circSpdyA and 127aa was inhibited, as shown by flow cytometry (Fig. 6F). These observations indicated that 127aa could disrupt the infiltration and cytotoxic function of NK cells in the in vitro microenvironment.

### 127aa inhibits the transcription of NK cell-activating factors

**Fig. 7:**
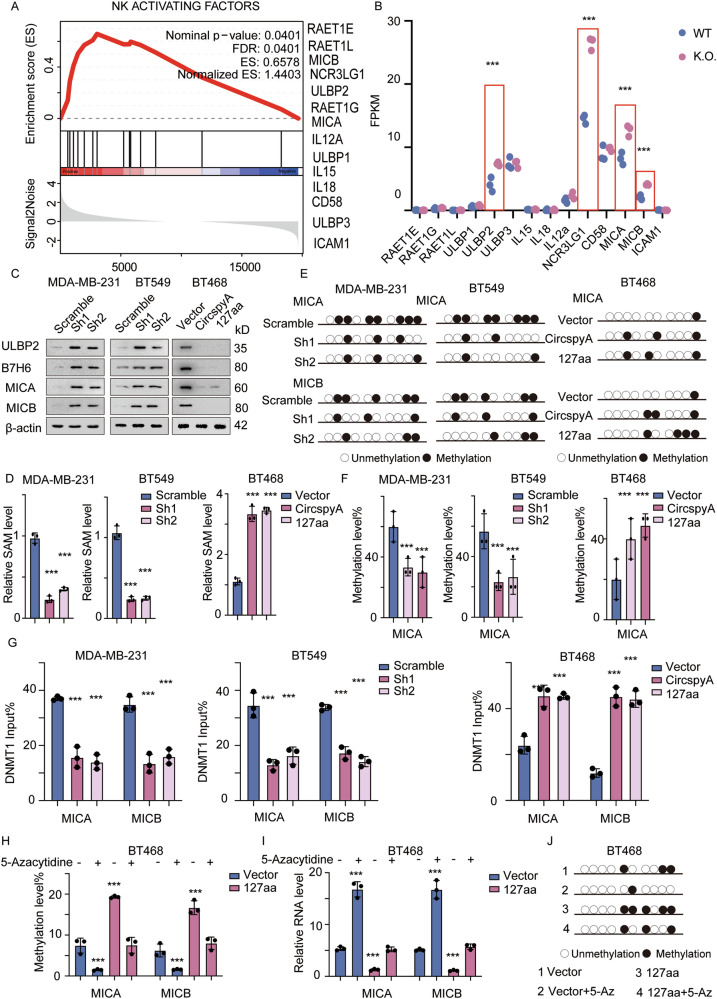
127aa inhibits the transcription of NK-activating factors. **A** circSpdyA knocking out cells were established and subjected to RNA sequence, GESA analysis was applied concerning the NK activating factor, and the activating factor was listed. **B** The FPKM of each activating factor in the RNA sequence. **C** The relative SAM level in cells with indicated modifications. **D** The immunoblot detects NK cell activating factors in cells with indicated modifications. **E** BSP analysis was applied to detect the methylation level of the promoter of MICA and MICB, the illustration image was presented. **F** The statistical analysis of the methylation of the promoters. **G** The engagement of DNMT1 on the promoter of MICA and MICB in cells with indicated modifications. **H**–**J** DNA methylation inhibitor 5-Azacytidine was applied to treat cells. The methylation rate was determined. The relative RNA level was detected and the BSP analysis was applied.

NK cell activation is regulated by a dynamic balance between complementary and antagonistic pathways. NK cells can perform effector functions when signaling is triggered by activating receptors instead of inhibitory receptors [20, 21]. To determine the potential mechanism through which 127aa affects NK cell activation, we established a circSpdyA-knockout MDA-MB-231 cell line using the CRISP-Cas9 system. The cells were harvested and subjected to RNA-seq, after which GSEA was applied to analyze potential NK activators (Fig. 7A). Among those activator factors, UL16 binding protein 2 (ULBP2), natural killer cell cytotoxicity receptor 3 ligand 1 (NCR3LG1), MHC class I polypeptide-related sequence A (MICA) and MICB were significantly upregulated in the KO group compared to the WT group (Fig. 7B). This was further confirmed at the protein level using an immunoblotting assay (Fig. 7C), which indicated that 127aa impaired transcriptional activator factors and caused NK cell dysfunction. To uncover the potential regulation mechanism, We first predicted the potential transcript factors regulating the expression of NK activators (https://guolab.wchscu.cn/hTFtarget/#!/) and identified CTCF, Myc and SPI1 as potential transcript factor. By analyzing the RNA sequence of 127aa knocking cells, alternative splice pattern was obtained, the alternative splice events and level of each variant were determined. We next overexpress the NK activators in 127aa knocking down cell lines and detect the half-life time of the mRNA. All the results showed no difference between cells (Supplementary Fig. 2A–C). When it comes to metabolic reprograming mediated gene regulation, S-adenosylmethionine (SAM) mediated DNA methylation was one of the most important mechanisms. We thus detect the level of SAM and DNA methylation status in different cells. Fortunately, the level of SAM decreased in CircSpdyA knocking down cells and increased in 127aa overexpression cells (Fig. 7D). We next applied bisulfite sequencing PCR (BSP) analysis. Representative methylation levels were determined, and statistical analysis was performed. Methylation of the MICA/MICB promoter was decreased in circSpdyA-knockdown cell lines but increased in 127aa-overexpressing cells (Fig. 7E, F, Supplementary Fig. 2D). As DNA methylation is mediated mainly through DNMTs, we next examined the involvement of different DNMTs using a ChIP assay. The results indicated that in circSpdyA-knockdown cells, more DNMT1 was recruited to the promoter regions of MICA and MICB (Fig. 7G, Supplementary Fig. 3A). Moreover, DNMT1 was detected using immunoblot, no significant difference was obtained, indicating that 127aa promotes the engagements of DNMTs to the promotor region without affecting the level of DNMTs (Supplementary Fig. 3B). A DNA methylation inhibitor-5-Azacytidine was applied in 127aa overexpression cells, the methylation level, relative RNA level was detected. As expected, 5-Azacytidine reversed 127aa mediated DNA methylation and NK activator suppression (Fig. 7H–J). These data suggested that circSpdyA inhibited the transcription of NK cell-activating factors.

### Fatty acid-laden TNBC cells inhibit NK cell function by paracrine signaling

**Fig. 8:**
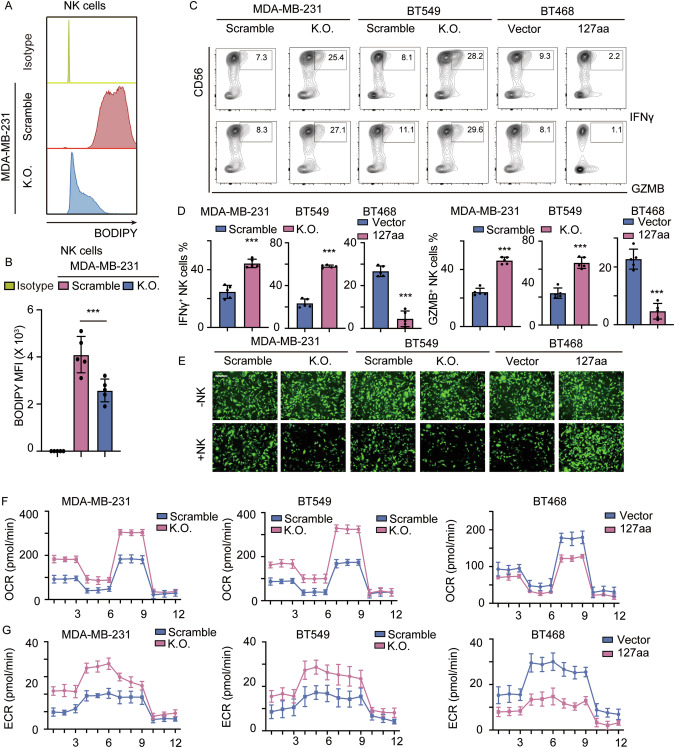
127aa mediated lipid-laden inhibits NK cell function. **A** MDA-MB-231 cells were stained with BODIPY and incubated with NK cells, the bodily in NK cells were detected using flow cytometry. **B** The statistical analysis of BODIPY intensity in NK cells is described in (**A**). **C** Flow cytometry analyzing the IFNγ^+^ GZMB^+^ proportion NK cells described in (**A**). **D** The statistical analysis of (**B**). **E** NK cells were incubated with circSpdyA KO and overexpression cells, NK cells were collected and co-cultured with cancer cells, Calcein-AM cell killing assay was applied, and the representative image was captured. **F**, **G** The OCR and ECR of NK cells are described in (**E**).

Studies have shown that human NK cell dysfunction in the tumor microenvironment occurs due to the inhibition of glucose metabolism through lipid peroxidation-related oxidative stress [22]. Lipid uptake and accumulation were proven to inhibit NK cell function. In addition, lipid-treated NK cells exhibit impaired effector functions and reduced tumor cytotoxicity in vivo [19]. To determine how alterations in lipid metabolism in tumors affect the functionality of NK cells, we established a cocultured system in which MDA-MB-231 and NK cells shared a medium but did not contact each other. MDA-MB-231 cells were stained with BODIPY. We measured the concentrations of BODIPYBODIPY in NK cells to determine the paracrine capacity of TNBC cells. The results demonstrated that the concentration of BODIPY was lower in NK cells that were cocultured with circSpdyA-knockout TNBC cells compared with scramble cells, suggesting that FAs may be translocated from FA-laden cancer cells to NK cells in a dose-dependent manner (Fig. 8A, B). NK cells cocultured with the indicated cancer cells were collected and subjected to flow cytometry to analyze the proportions of CD56-, IFNγ-, and granzyme B (GZMB)-positive NK cells. NK cells with high lipid levels exhibited impaired cytotoxicity (Fig. 8C, D). NK cells cocultured with the indicated tumor cells were collected and subsequently cocultured with MDA-MB-231 cancer cells. The results demonstrated that specific NK cell-mediated target cell killing dramatically decreased when NK cells had higher lipid levels (Fig. 8E). These results suggested that lipids released from cancer cells inhibited NK functions. For further confirmation, NK cells cocultured with the indicated cancer cells were collected and subjected to OCR and extracellular acidification rate (ECR) assays to assess metabolic reprogramming. The function of NK cells in the antitumor response requires the reprogramming of glucose-driven glycolysis and mitochondrial oxidative phosphorylation (OXPHOS). Increased mitochondrial respiration and decreased glycolysis are observed when NK cells exhibit cytotoxicity in the TME [22, 23]. As expected, NK cells with increased lipid levels had a low OCR but a high acidification rate (Fig. 8F, G). Together, our analyses indicated that FA-laden tumor cells could inhibit NK function via paracrine signaling.

**Fig. 9:**
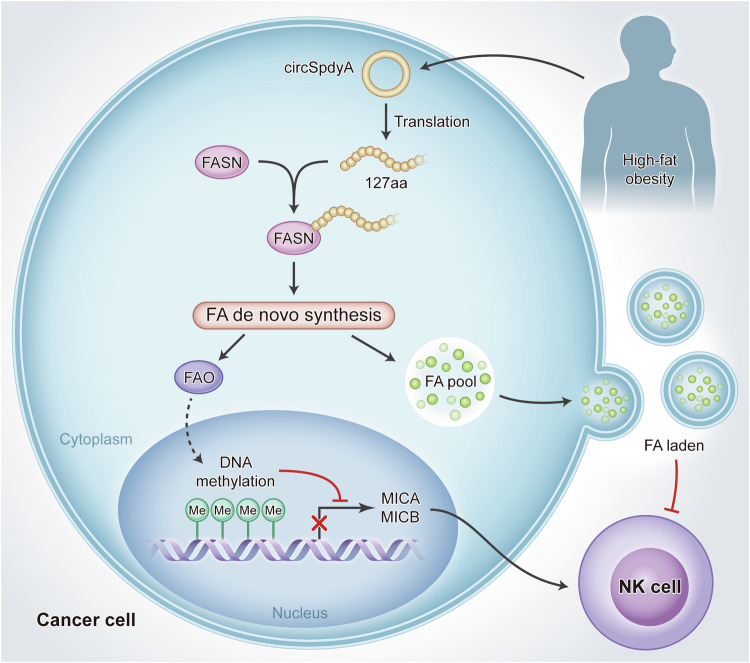
Graphic abstract of 127aa on lipid metabolism reprogram and NK repression. circSpdyA was induced by high-fat obesity, 127aa was translated using circSpdyA as a template, 127aa directly binds with FASN and promotes FA de novo synthesis, enhanced FAO promotes the DNA methylation of NK activators such as MICA and MICB, which finally inhibited NK cell infiltration. Additionally, excess FA pool produced FA laden and paracrined to NK cells to inhibit NK cell function.

Taken together, the graphic abstract of 127aa on FA metabolism reprogram and NK suppression was illustrated in Fig. 9.

## Discussion

The lack of specific targets limits the treatment of triple-negative breast cancer, and this is a challenging medical problem. In this study, we provided mechanistic evidence that a novel peptide, 127aa, encoded by circSpdyA could regulate FA metabolism in TNBC, leading to tumorigenesis and inhibition of NK cell function. Our results revealed that overexpressing circSpdyA or 127aa induced FA synthesis through their direct binding to FASN, thus increasing FA accumulation. In addition, 127aa inhibited the NK cell activating factors ULBP2, NCR3LG1, MICA, and MICB by controlling their transcription, thus resulting in dysfunction and decreased infiltration of NK cells in the TME. In vivo, loss of circSpdyA led to a reduction in tumor growth. Simultaneously, immune checkpoint blockade (ICB) therapy increased effector NK cell function. These findings support the further development of approaches that target 127aa in TNBC to enhance antitumor effects by inhibiting FA metabolism and improving the NK cell-mediated immune response.

For more than a decade, many comprehensive trials have made breakthroughs in classifying TNBC subtypes by conducting multiomics analyses for integrating comparisons [24]. The FUTURE trial is well known and revealed four subtypes related to genomics and transcriptomics, three subtypes related to metabolic pathway-based heterogeneity, and three subtypes related to comprehensive metabolomics [1, 2, 4]. The metabolome C1 subtype, which is characterized by the enrichment of ceramides and fatty acids, attracted our attention due to its overlap with the LAR transcriptome subtype. A previous study demonstrated that the LAR subtype is characterized by activated ferroptosis-related pathways, such as FA synthesis, and is vulnerable to ferroptosis. These findings further indicated that GPX4 is an essential metabolic target and that the combination of GPX4 inhibitors and ICB may be an effective strategy for treating this TNBC subtype [5]. The reactivation of de novo FA synthesis results in high metabolic pressure on cancer cells, indicating that reprogrammed FA metabolism contributes to tumorigenesis and progression [25]. Fatty acid synthesis and oxidative metabolism are two interrelated processes. Abnormal fatty acid oxidation (FAO) is an important component of tumor energy metabolism reprogramming. Response to nutrient stress, cancer cells may tend to use fatty acid as an energy source, and de novo synthesis will in turn activated, FAO ensures the plasticity of metabolic reprogramming by producing ATP and NADPH, eliminating potential toxic lipids, inhibiting pro apoptotic pathways, and providing metabolic intermediates for tumor cell growth (PMID: 23446547). When it comes to fatty acid synthesis, the activation of fatty acid synthesis will produce an excess of Fatty acid, which serves as an important material for the synthesis and maintenance of cell membranes. Additional fatty acid will promote the FAO to produce energy and one-carbon unit for biosynthesis. It was previously proven that FA synthesis is enhanced in breast tumor metastases in the brain. The key enzyme involved in FA synthesis, FASN, is associated with a higher risk of cancer recurrence and mortality. The inhibition of FASN could inhibit the growth of brain metastases in patients with breast cancer, suggesting that FASN is a potential therapeutic target for treating breast cancer metastasis [3, 26]. Studies have shown that RARRES2 deficiency in cancer cells promotes TNBC brain metastasis by altering lipid metabolism in the brain microenvironment [27]. Inhibiting FAO decreases energy metabolism in MYC-overexpressing TNBC cells and inhibits tumor growth in a patient-derived xenograft (PDX) model [28]. However, there is still a lack of research on the potential factors that regulate FA metabolism in the primary foci of TNBC. Our work revealed an essential circRNA, circSpdyA, and a crucial peptide, 127aa, that link FA metabolic reprogramming and the development of TNBC, 127aa binds with FASN at 1792 residue, located in ER domain, with catalytical activity. Our research indicated that the interaction between 127aa and FASN promotes the activity of de novo Fatty acid synthesis. This may be due to that 127aa promotes the activity of ER domain.

The biological functions of circRNAs in tumorigenesis and TME regulation have attracted increasing amounts of attention. CircRNAs can interact with lipid metabolism in cancer. For instance, circCAPRIN1 enhances the regulation of lipid metabolism and facilitates tumor progression in colorectal cancer [29]. circLARP1B promotes lipid accumulation and invasion in hepatocellular carcinoma (HCC) through a conserved mechanism in mammals. circPRKAA1 promotes de novo FA synthesis and maintains lipid homeostasis in HCC. Both of these factors are closely associated with poor prognosis in HCC patients [11, 30]. circMBOAT2 was found in intrahepatic cholangiocarcinoma tissues and can facilitate FASN mRNA cytoplasmic export and altered lipid metabolic activity [12]. Emerging evidence has suggested that circRNAs, as noncoding RNAs, can also translate short peptides, thereby promoting cancer cell proliferation, survival, and invasion [6, 31]. Moreover, circRNAs were shown to function by coding proteins that facilitate oncogenesis and aggression in TNBC. The protein-coding gene circ-EIF6 was shown to promote TNBC cell proliferation and metastasis in vivo and in vitro by encoding EIF6-224aa, which can decrease MYH9 degradation and activate the Wnt/beta-catenin pathway [32]. CircSEMA4B was proven to be downregulated in breast cancer tissues and to play a negative regulatory role in the PI3K/AKT signaling pathway by encoding SEMA4B-211aa [33]. One recent study revealed that a novel polypeptide, CAPG-171aa, is encoded by circCAPG and activates the tumor growth pathway MEKK2-MEK1/2-ERK1/2 [34]. However, little is known about the regulatory role of peptides encoded by circRNAs in FA metabolic reprogramming and the immune microenvironment in TNBC. Herein, we found that circSpdyA and its encoded 127aa were upregulated in TNBC. These factors play imperative roles in the TME by promoting cancer cell growth by enhancing de novo FA synthesis and disrupting the antitumor capacity of NK cells both in vitro and in vivo.

FA-mediated metabolic reprogramming in breast cancer not only provides energy for the proliferation and migration of tumor cells but also weakens the antitumor immune system in the TME. Altering or accumulating metabolic substances might impair the ability of immune cells in the TME to recognize antigens and initiate immune responses to tumors. Increasing lipid accumulation and uptake has been reported to induce immune cell dysfunction in the TME, such as inhibiting the antigen-presenting capacity of dendritic cells and impairing the mitochondrial function of CD8^+^ T cells [35]. Furthermore, FA metabolism remodeling in the TME could serve as an attractive target and lead to improved combinatorial treatment strategies for immune therapy. A study showed that CD8^+^ T cells increase their FA metabolism to meet energy needs and exert stronger antitumoural immune effects when combined with the PD-L1 antibody in the TME [36]. During tumor-associated macrophage (TAM) polarization toward the M2-like phenotype (protumoral), peroxisome proliferator-activated receptor-γ (PPARγ) and CD36 are upregulated, and FA uptake and oxidation are promoted. Several studies have shown that targeting FA metabolism in TAMs can inhibit M2 polarization in vitro and effectively control tumor growth in vivo [37]. Targeting the interaction between FA metabolism and immune cells in the TME holds tremendous potential prospects for treatment. In the present study, we showed that the 127aa sequence not only played a role in the activation of fatty acid synthesis by binding to FASN but also interfered with NK cell function by inhibiting transcription of NK cell-activating factors. Accordingly, these findings might shed light on the 127aa-mediated epigenetic mechanism by which NK cells are rendered dysfunctional by disordered metabolism.

NK cells are among the requisite immune cells in the TME. Intriguingly, in an oxidative stress microenvironment caused by lipid peroxidation and FA, these cells attenuate their cytotoxicity and enhance their lipid metabolism to survive. Obesity induces PPAR-driven lipid accumulation in NK cells and impairs the mTOR signaling pathway, inhibiting intracellular transport and leading to cytotoxic defects [19]. In the pre-lung-metastatic stage of BC, lung mesenchymal cells undergo metabolic alterations by accumulating intercellular lipids and transferring them to tumor cells and NK cells. This process inhibits the function of NK cells and ultimately promotes the occurrence of metastasis [38]. Jiao et al. reported that FAs could induce the sustainable dysfunction of NK cells by disrupting the c-Myc/P300/H3K27ac axis. Forced c-Myc expression restored the cytokine-producing and antitumor activity of NK cells in the TME [39]. Here, we elucidated a previously unknown mechanism underlying the interaction between FA-laden tumor cells and NK cell inhibition in vitro. The expression of 127aa could result in different levels of metabolic excretion in tumor cells, followed by stepwise changes in metabolic pathways in NK cells and, in turn, NK dysfunction, as shown by the decreases in CD56, IFNγ, and GZMB expression.

In conclusion, our study emphasizes that a novel peptide, 127aa, which is encoded by circSpdyA in TNBC, promotes tumorigenesis by enhancing fatty acid synthesis and impairs NK cell infiltration and function via the reprogramming of fatty acid metabolism. Importantly, a potential strategy for targeting 127aa was explored to improve NK cell efficacy and optimize immune checkpoint blockade therapy. However, there are still limitations to our study. We did not thoroughly investigate how 127aa interferes with specific activating factors that can affect the effector function of NK cells in antitumor immunity. Moreover, the detailed signaling pathways by which 127aa and FA metabolism regulate NK function in the TME have yet to be identified. These underlying aspects need to be further investigated.

**Table:** 

Star methods		
REAGENT or RESOURCE	SOURCE	IDENTIFIER
**Antibodies**		
Anti-Flag	Thermo Fisher Scientific	MA1-91878
Anti-β-actin	Cell signaling technology	Cat #4967
Anti-PCNA	Cell signaling technology	Cat #13110
Anti-N-caherin	Cell signaling technology	Cat #13116
Anti-E-caherin	Cell signaling technology	Cat #3195
Anti-Vimentin	Cell signaling technology	Cat #5741
Anti-Zeb1	Cell signaling technology	Cat #3396
Anti-ACC	Cell signaling technology	Cat #3662
Anti-FASN	Cell signaling technology	Cat #3180
Anti-SCD	Cell signaling technology	Cat #2794
Anti-CD56	BD Pharmingen™	567479
Anti-IFN-γ	BD Pharmingen™	557735
Anti-ULBP2	Cell signaling technology	Cat #15329
Anti-NCR3LG1	Cell signaling technology	Cat #65566
Anti-MICA	Cell signaling technology	Cat #81875
Anti-MICB	Cell signaling technology	Cat #77296
Anti-GZMB	BD Pharmingen™	561999
Anti-Aurora A	Cell signaling technology	Cat #14475
**Recombinant DNA**		
psPAX2	Addgene	N/A
pMD2G	Addgene	N/A
**Dataset**		
CircRNA sequence (OD VS HD)	This study	HRA006478
ScRNA sequence	This study	HRA006479
RNA sequence (WT VS circSpdyA KO)	This study	HRA006477
**Biological Samples**		
Human breast cancer tissues	Guangzhou Women and Children’s Medical Center	N/A
**Chemicals, peptides, and recombinant proteins**		
Escherichia coli transfer RNA	Life Technologies (Thermo Fisher Scientific)	N/A
Salmon sperm DNA	Life Technologies (Thermo Fisher Scientific)	N/A
BSA	Roche (Shanghai, CHN)	N/A
Fluorescently labeled junction probe	Generay (Shanghai, CHN)	N/A
ProLong^TM^ Gold	Life Technologies (Thermo Fisher Scientific)	N/A
PVDF membrane	Millipore (Massachusetts, USA)	N/A
Lipofectamine™ 3000	Thermo Fisher scientific	N/A
Polybrene	Sigma	N/A
**Critical commercial Assays**		
VAHTS Total RNA-seq (H/M/R) Library Prep Kit for Illumina	Vazyme (Nanjing, CHN)	N/A
PrimeScript^TM^ RT Master Mix	Takara	RR036
TB Green^®^ Premix Ex Taq^TM^ II	Takara	RR820
Cell Counting Kit-8	Dojindo (Tabaru, Japan)	Cat# 96992
Calcein-AM staining kit	Solarbio	CA1630
**Experimental Models: Cell lines**		
Human breast cancer cell MDA-MB-231	ATCC	Cat# HTB-26; RRID: CVCL_0062
Human breast cancer cell MCF-7	ATCC	Cat# HTB-22; RRID: CVCL_0031
Human breast cancer cell Hs598T	ATCC	Cat# HTB-126
Human breast cancer cell MDA-MB-453	ATCC	Cat# HTB-131; RRID: CVCL_0418
Human breast cancer cell MDA-MB-468	ATCC	Cat# HTB-132; RRID: CVCL_0419
Human breast cancer cell BT549	ATCC	Cat# HTB-122; RRID: CVCL_1092
Human natural killer cell NK-92MI	ATCC	Cat# CRL-2408
Human embryonic kidney cell line HEK293T	ATCC	Cat# CRL-3216; RRID: CVCL_0063
Mouse breast cancer cell 4T1	ATCC	Cat# CRL-2539
**Experimental Models: Organism/Strains**		
Female athymic (Ncr nu/nu) mice (6 to 8-week-old)	Nanjing University Farms	N/A
Female C57BL/6 mice (6 to 8-week-old)	Nanjing University Farms	N/A
**Others**		
circRNA sequencing	Gene Denovo Biotechnology Co. (Guangzhou, CHN)	N/A
OmicShare tools	Gene Denovo Biotechnology Co. (Guangzhou, CHN)	www.omicshare.com/tools
Flow cytometry	BD FACSymphony^TM^ A3	N/A
Confocal microscope	FV100 (Olympus FluoView^TM^)	N/A
QuantStudio^TM^ 5	Applied Biosystems (Thermo Fisher Scientific)	N/A
Q Exactive mass spectrometer	Thermo Fisher Scientific	N/A
RCSB Protein Data Bank	US	www.rcsb.org
The ClusPro server	Vajda Lab and ABC Group (Boston University and Stony Brook University, US)	cluspro.org
PyMOL	the Schrodinger Sales Center	pymol.org
Human Metabolome Database (HMDB)	Canada	www.hmdb.ca
MassBank	Japan	www.massbank.jp
LipidMaps	US	www.lipidmaps.org
mzCloud	Slovakia	www.mzcloud.org
Kyoto Encyclopedia of Genes and Genomes	Japan	www.genome.jp/kegg
Ropls	Bioconductor	N/A
MetaboAnalyst	JavaServer Faces Technology	www.metaboanalyst.ca

## Materials and methods

### Xenograft studies

Six to 8-week-old athymic (NCr nu/nu) and C57BL/6 female mice were obtained from Nanjing University Farms in this study. For in situ xenograft, 5×10^5^ breast cancer cells were transplanted into mice subcutaneously. Mice were treated with an ordinary diet or a high-fat diet. For lung metastasis assay, 1 ×10^5^ cells were injected, and mice were sacrificed six weeks post-injection. The number of lungs with surface metastasis were determined, as well as the number of surface metastases per lung by examination under a dissecting microscope.

For CTCs detection, cancer cells were tagged with GFP before injection, after vein injection and 6-week feeding, we took blood from the hearts of mice, about 0.5–1 ml. Blood samples are quickly mixed with EDTA to prevent blood clotting. Centrifuge at 800–1000 g for 10 min to remove the plasma and retain the cell precipitate. The cells were then treated with red blood cell lysate to remove the red blood cells and obtain mononuclear cells (PBMCs). CTCs were then enriched for positive selection, CTCs were sorted using FACS by isolating GFP positive cells. Negative selection was then performed to indirectly enrich CTCs by removing cells expressing the leukocyte marker CD45. CTCs were quantified by normalizing to CD45. C57BL/6 mice were transplanted with 127aa-overexpressed 4T1 cells subcutaneously, samples were collected and subjected to single cell sequence.

### circRNA sequence

Samples from mice fed with ordinary diet and high-fat diet were collected. Total RNAs were extracted, digestive, and purified. Next, a strand-specific library was constructed using a VAHTS Total RNA-seq (H/M/R) Library Prep Kit from Illumina (Vazyme, Nanjing, CHN) according to the manufacturer’s instructions. After removing the ribosome RNAs. The enriched circRNAs were fragmented into short fragments through a fragmentation buffer and reverse transcribed into cDNA with random primers. The cDNA fragments were purified with VAHTSTM DNA Clean Beads, then end-repaired, supplemented with poly(A), and ligated to Illumina sequencing adapters. Then, the digested products were purified with VAHTSTM DNA Clean Beads, amplified by PCR, and sequenced using Illumina HiSeq^TM^ 2500 by Gene Denovo Biotechnology Co. (Guangzhou, China). After aligning with the reference genome, the reads that could be mapped to the genomes were discarded, and the unmapped reads were then collected for circRNA identification. Then, 20mers from both ends of the unmapped reads were extracted and aligned to the reference genome to find unique anchor positions within the splice site. Differential expression analysis of the RNA-seq was performed by edgeR in the OmicShare tools, a free online platform for data analysis (www.omicshare.com/tools). The default parameters of edgeR were utilized, and Differential expression genes (DEGs) were selected according to log2 fold change >=1 and p value < 0.05.

### Flow cytometry

For detection of surface markers, samples were prepared and resuspended in a staining buffer, after incubation with fluorescence-labeled primary antibody. Samples were analyzed for flow cytometry on BD FACSymphony™ A3. For assessment of lipids, NK cells were stained with Bodipy reagents and measured by flow cytometry.

### RNA fluorescence in situ

Cells were incubated at 37 °C in a solution containing 50% formamide, 2× SSC, 0.25 mg ml^−1^ Escherichia coli transfer RNA, 0.25 mg ml^−1^ salmon sperm DNA (Life Technologies), 2.5 mg ml^−1^ BSA (Roche) and 125 nM fluorescently labeled junction probe (Generay). After 12 hours, the cells were washed with cold PBS 2-3 times and sealed with a ProLong^TM^ Gold mounting media (Life Technologies) then incubated overnight at room temperature. Confocal images were visualized with the Olympus FV100 confocal microscope.

### qRT-PCR

RNA was reverse transcribed using PrimeScript RT Master Mix (RR036, Takara) according to the manufacturer’s protocol. qPCR was conducted with TB Green® Premix Ex TaqTM II (Tli RNaseH Plus) (RR820, Takara) in a QuantStudio 5 system (Applied Biosystems).

### Tumor specimen and ethical approval

Human breast tumor tissues were obtained from the Department of Breast and Thyroid Surgery of Guangzhou Women and Children’s Medical Center. All clinical samples were collected with the informed and signed consent of each patient. The study adhered to the Ethics Institutional Review Boards of the above institutes. The data was compliant with all relevant ethical regulations regarding research involving human participants.

### LC-MS analysis

The immunoprecipitates were harvested and digestion was performed. Mass spectrometry detection of the resulting peptide was carried out on a QExactive mass spectrometer coupled to a nano-LC (AdvanceLC). The acquired spectra were analyzed using the SEQUEST HT algorithm.

### Western blot

The protein lysates extracted from cultured cells or patient tissues were loaded into a 12% SDS-PAGE gel followed by electrophoresis. The gels were transferred to the PVDF membrane (Millipore, Massachusetts, USA). The membranes were first blocked with 5% milk and then incubated with the indicated primary antibody, followed by the conjugated secondary antibody tagged with horseradish-peroxidase. Enhanced chemical luminescence assays were performed to visualize the signals.

### Immunohistochemistry staining

Breast cancer tissue sections (8–10 μm thick) were deparaffinized and blocked. The sections were incubated with primary antibodies diluted in bovine serum albumin overnight at 4 °C in a wet chamber. After incubation in secondary antibodies, chromogenic immunolocalization was performed using the diaminobenzidine reagent. These slides were then counterstained with hematoxylin to visualize nuclei. The immunoreactivity of antigen was measured based on the staining intensity and proportion of positive cells. The evaluated values of the staining intensity and the stained tumor cells percentage resulted in the scores, which multiplied ranging from 0 to 12, whereby 0–6 was considered low expression and 7–12 was considered overexpression.

### Lentiviral production and establishment of stable cell lines

Lentiviral vectors were conducted with the expressions of shRNA, circSpdyA, 127aa, 127aa-mut, FASN, and FASN-mut, then co-transfected with the packaging vectors psPAX2 (Addgene) and pMD2G (Addgene) into HEK293T cells for lentivirus production using Lipofectamine 3000 (Thermo Fisher Scientific) according to the manufacturer’s instructions. To establish stable cell lines, cancer cells were transduced with these above lentiviruses with the addition of 8 mg ml^−1^ Polybrene (Sigma). After 72 h of incubation, cells were selected with 2 mg ml^−1^ puromycin for 3 days.

### Cell viability (CCK-8 and colony formation assay)

For measurement of proliferation, Cell Counting Kit-8 (CCK-8; Dojindo, Tabaru, Japan) was utilized to determine cell viability by sensitive colorimetric assays. Two hundred cells/well were seeded in 96-well plates. The absorption values were collected at 24, 48, and 72 h after shRNA transfection. The experiments were repeated three times. The data are shown as the mean ± standard deviation (SD). To assess the ability of cell division, transfected BC cells were seeded in a 6-well plate at a density of 500 cells/well. After 2 weeks of culture, cell colonies were fixed with 4% paraformaldehyde and stained with 1% crystal violet. Counted the number of colonies using a stereomicroscope.

### Molecular docking

The 3D structure of the FASN protein was downloaded from the RCSB Protein Data Bank. Protein–protein docking in ClusPro server4-8 (https://cluspro.org) was used for molecular-docking simulations of 127aa and for predicting the binding affinity to FASN. Molecular graphics were generated using PyMOL.

### Immunoprecipitation

Cells were lysed in ice-cold lysis buffer (0.3% CHAPS, 10 mM β-glycerol phosphate, 10 mM pyrophosphate, 40 mM HEPES (pH 7.4), 2.5 mM MgCl_2_ and EDTA-free protease inhibitor). The soluble fractions from cell lysates were immunoprecipitated using Neat-31 antibody or Aurora A. Then, the mix was rotated overnight at 4 °C. Beads were added and incubated for 2 hours at room temperature. Immunoprecipitates were washed three times with PBST and subjected to immunoblotting and LC-MS analyses.

### Calcein AM cytotoxicity assay

This assay was performed using a calcein AM staining kit (CA1630, Solarbio) according to the manufacturer’s protocols. Briefly, cancer cells (1 × 10^5^ cells/ml) were seeded into 96-well plates. 1 μM calcein AM in culture medium was added to the cells and was then incubated for 15 min at 37 °C. Subsequently, NK-92MI or primary NK cells were seeded into the wells at a density of 0.5^−1^ × 10^6^ cells/ml to achieve a 1:5 or 1:10 ratio. The cells were cocultured for 2 h. Images were acquired with a microscope.

### LC-MS metabolites analysis

The metabolites were identified by accuracy mass (< 30 ppm) and MS/MS data which were matched with HMDB (http://www.hmdb.ca), massbank (http://www.massbank.jp/), LipidMaps (http://www.lipidmaps.org), mzclound (https://www.mzcloud.org) and KEGG (http://www.genome.jp/kegg/).

The Ropls software was used for all multivariate data analyses and modelings. After scaling data, models were built on principal component analysis (PCA), orthogonal partial least-square discriminant analysis (PLS-DA), and partial least-square discriminant analysis (OPLS-DA).

The metabolic profiles could be visualized as a score plot, where each point represents a sample. OPLS-DA allowed the determination of discriminating metabolites using the variable importance on projection (VIP). The P value, variable importance projection (VIP) produced by OPLS-DA, and fold change (FC) were applied to discover the contributable variable for classification. Finally, P value < 0.05 and VIP values > 1 were considered to be statistically significant metabolites. Differential metabolites were subjected to pathway analysis by MetaboAnalyst, which combines results from powerful pathway enrichment analysis with the pathway topology analysis. The identified metabolites in metabolomics were then mapped to the KEGG pathway for biological interpretation of higher-level systemic functions. The metabolites and corresponding pathways were visualized using the KEGG Mapper tool.

## Supplementary information

supplementary figure

## Data Availability

All data needed to evaluate the conclusions in the paper are present in the paper and/or the Supplementary Materials. CircRNA sequence (OD VS HD), HRA006478; ScRNA sequence, HRA006479; RNA sequence (WT VS circSpdyA KO), HRA006477. Additional data related to this paper may be requested from the authors. All the uncut wb blot was shown in supplementary Fig. 4.
