# Supplementary material for: 127aa encoded by circSpdyA promotes FA synthesis and NK cell repression in breast cancers
Source: Cell Death Differ. 2024 Oct 14;32(3):416–33. doi: 10.1038/s41418-024-01396-1 (PMC11894148; doi:10.1038/s41418-024-01396-1)
Supplement: Supplementary file 1 — supplementary figure [file 41418_2024_1396_MOESM1_ESM.pdf]

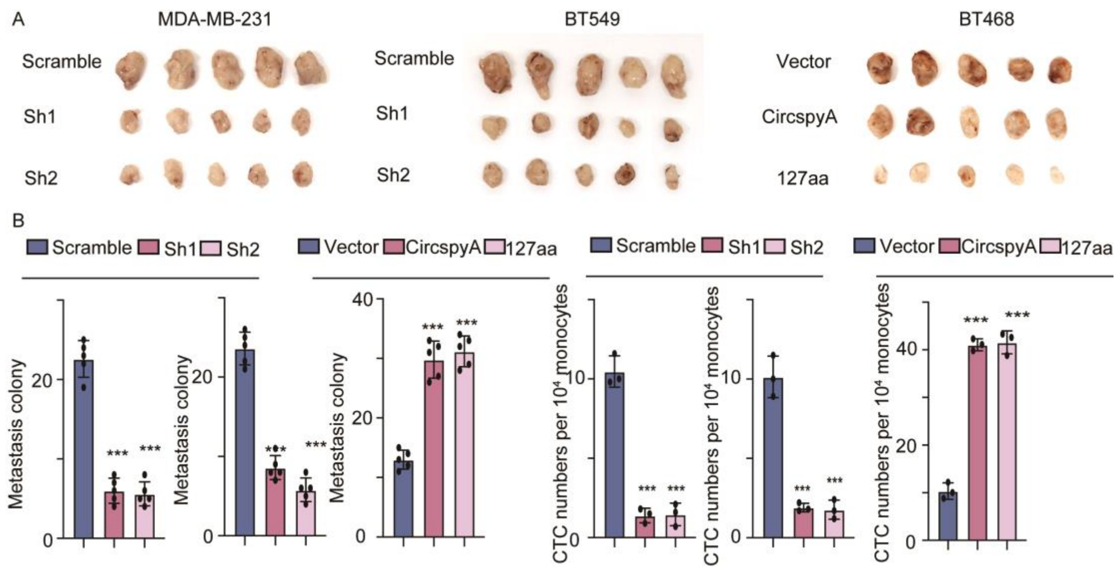


**Supplementary Figure 1. 127aa promotes tumor progression and metastasis.**

**A.** The picture of xenograft models.

**B.** The statistical analysis of lung metastasis colony and CTC numbers.

A

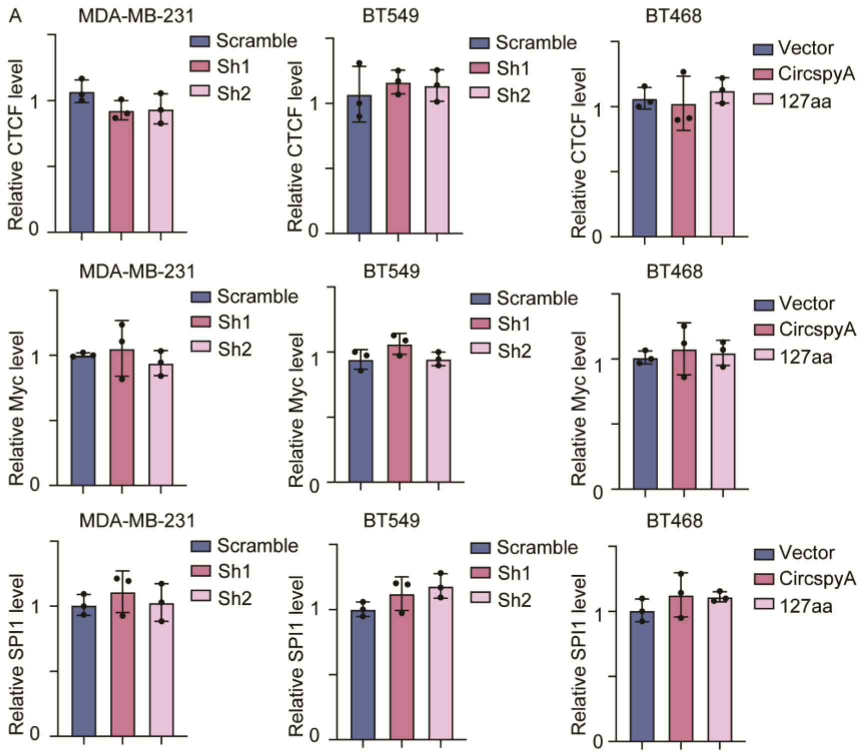


B

MDA-MB-231

Scramble VS Sh1

| | SE | RI | MXE | A5SS | A3SS |
|---|---|---|---|---|---|
| ULBP2 | NS | NA | NA | NS | NA |
| B7H6 | NA | NA | NA | NA | NA |
| MICA | NS | NA | NA | NS | NA |
| MICB | NS | NA | NA | NS | NA |

C

| | MDA-MB-231 | | | BT549 | | | BT468 | | | |
|---|---|---|---|---|---|---|---|---|---|---|
| | Sc | Sh1 | Sh2 | Sc | Sh1 | Sh2 | Vector | 127aa | CircspyA | |
| ULBP2 | 65 | 67 | 64 | 66 | 64 | 62 | 78 | 72 | 71 | |
| B7H6 | 54 | 56 | 50 | 56 | 58 | 49 | 61 | 54 | 64 | |
| MICA | 58 | 62 | 57 | 62 | 64 | 64 | 71 | 74 | 73 | |
| MICB | 61 | 63 | 58 | 64 | 67 | 66 | 74 | 76 | 78 | min |

D

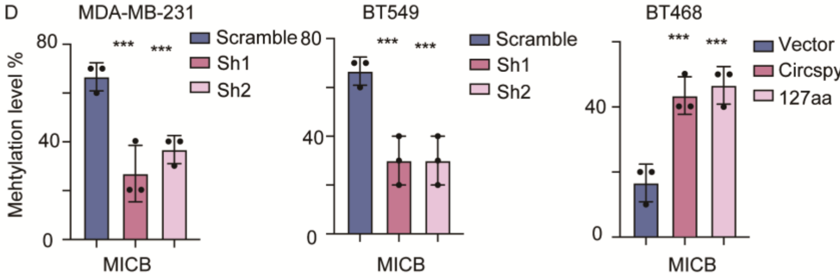


**Supplementary Figure 2. 127aa promotes the DNA methylation of NK activator.**

**A.** The relative level of potential transcript factor in cells with indicated modifications.

**B.** The alternative splicing of ULBP2/B7H6/MICA/MICB in The picture of xenograft models.

**C.** The half-life time of each gene in different cells.

**D.** DNA methylation of MICB in cells with different modifications.

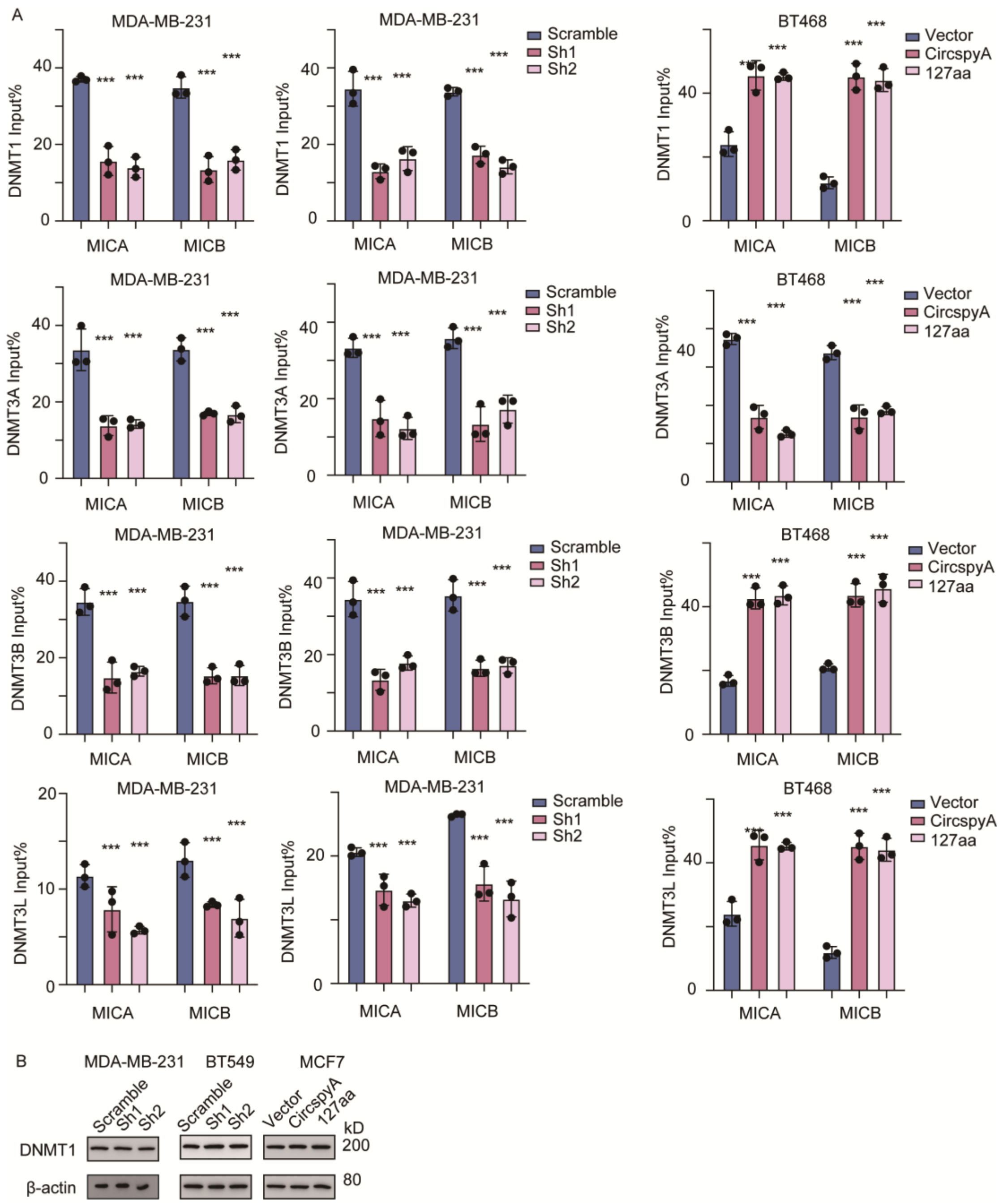


**Supplementary Figure 3. 127aa promotes the DNMTs recruitments to DNA promotor.**

**A.** The engagement of different DNMTs onto the DNA promotor.

**B.** Immunoblot detecting DNMT1 in cells with indicated modifications.

2D

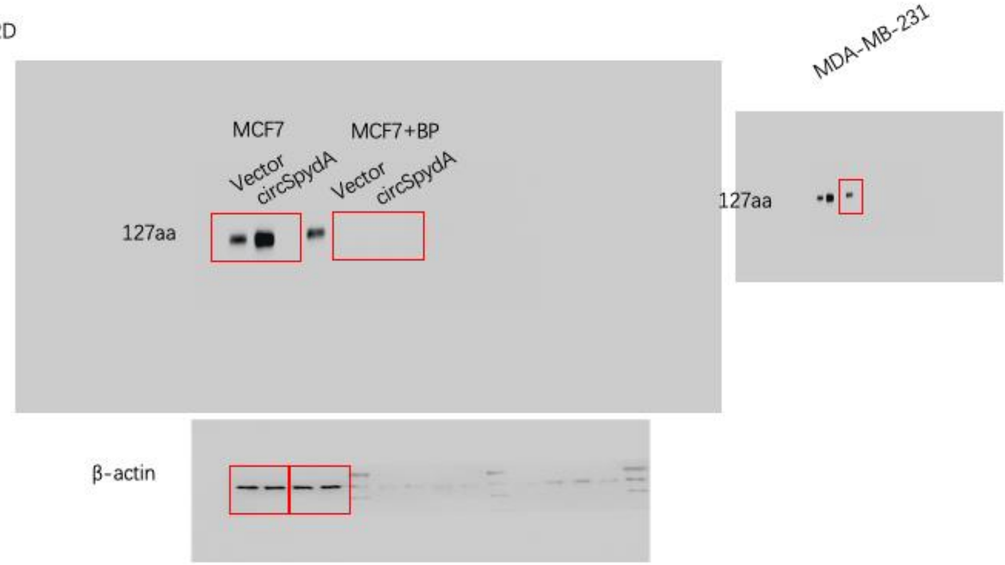
MCF7
MCF7+BP
Vector
circSpydA
Vector
circSpydA
127aa
MDA-MB-231
127aa
β-actin

2E

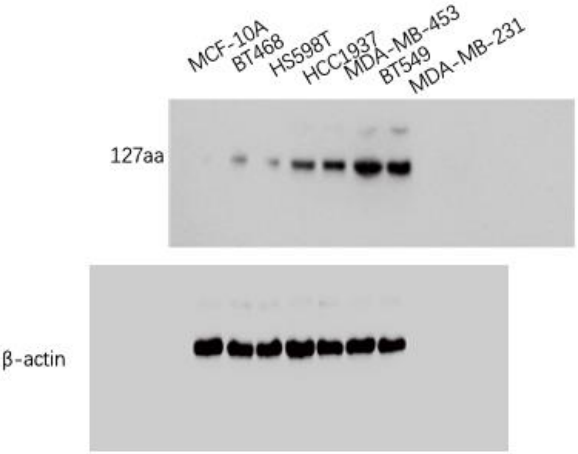
MCF-10A
BT468
HS598T
HCC1937
MDA-MB-453
BT549
MDA-MB-231
127aa
β-actin

2E

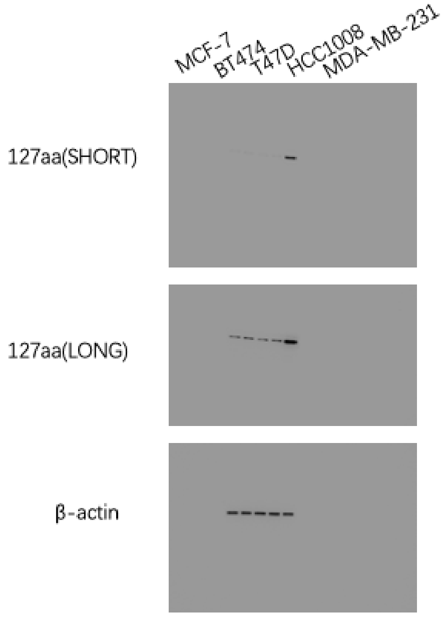
MCF-7
BT474
T47D
HCC1008
MDA-MB-231
127aa(SHORT)
127aa(LONG)
β-actin

2F

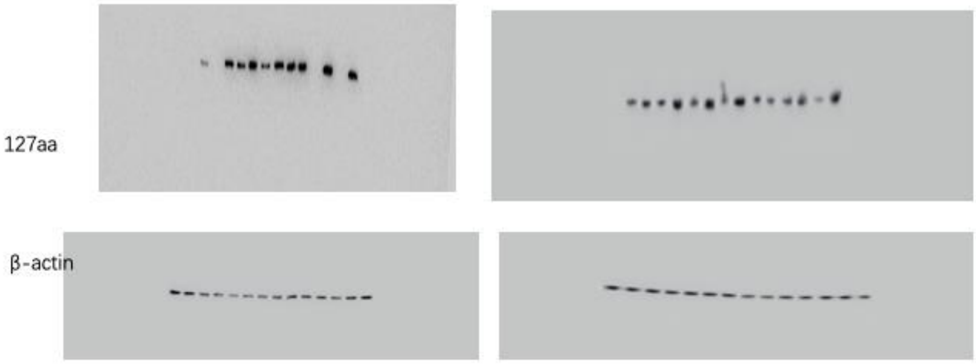
127aa
β-actin

3G

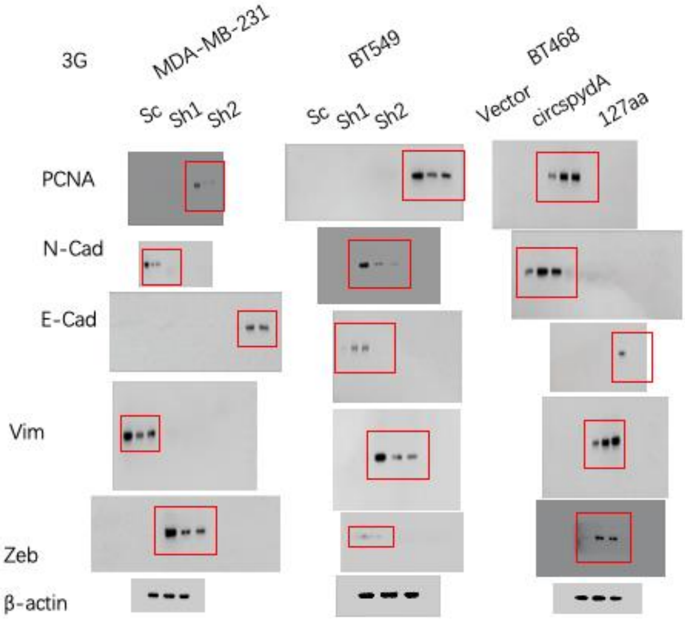
MDA-MB-231
BT549
BT468
Sc Sh1 Sh2
Sc Sh1 Sh2
Vector circspydA 127aa
PCNA
N-Cad
E-Cad
Vim
Zeb
β-actin

5B

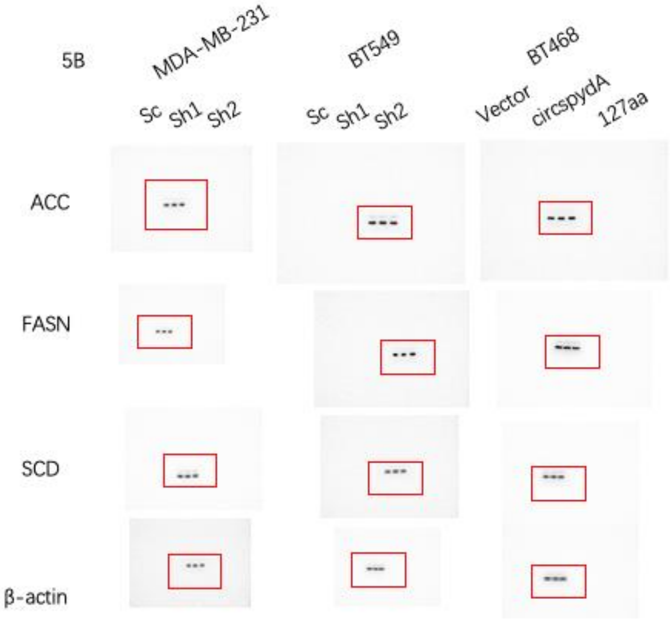
MDA-MB-231
BT549
BT468
Sc Sh1 Sh2
Sc Sh1 Sh2
Vector circspydA 127aa
ACC
FASN
SCD
β-actin

5D

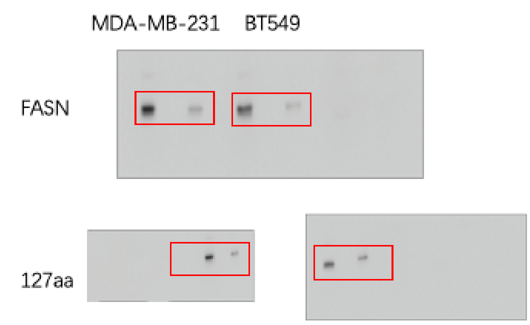
MDA-MB-231
BT549
FASN
127aa

5H

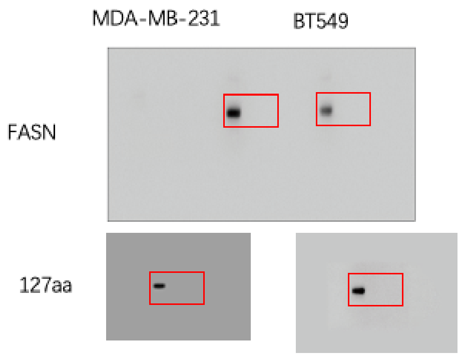
MDA-MB-231
BT549
FASN
127aa

5J

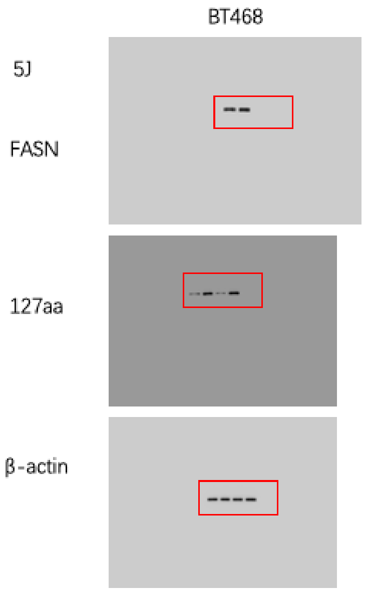
BT468
FASN
127aa
β-actin

5L

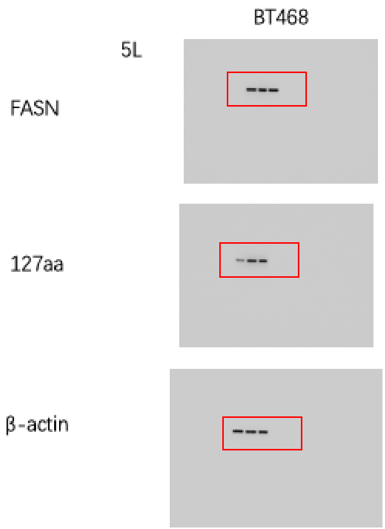
BT468
FASN
127aa
β-actin

5O

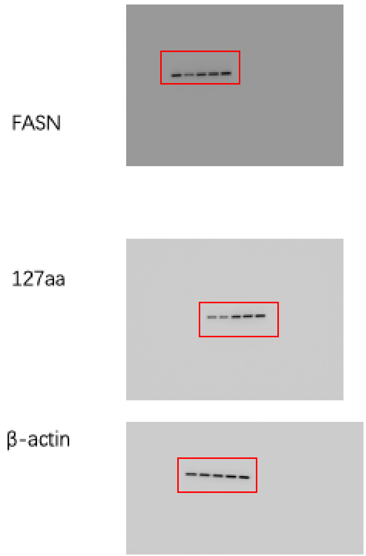
FASN
127aa
β-actin

7C

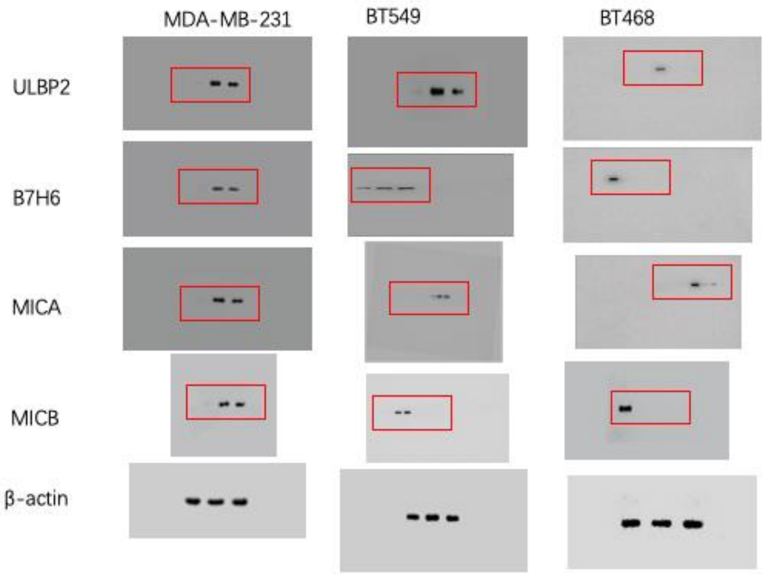
MDA-MB-231
BT549
BT468
ULBP2
B7H6
MICA
MICB
β-actin

S3

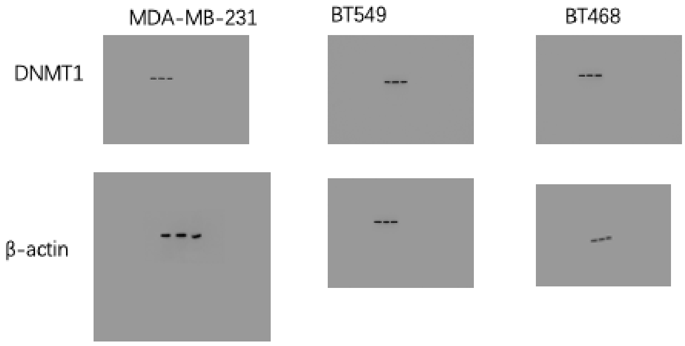


**Supplementary Figure 4. The uncut immunoblot image.**
